# Biological properties and surgical applications of the human amniotic membrane

**DOI:** 10.3389/fbioe.2022.1067480

**Published:** 2023-01-09

**Authors:** Jose R. Munoz-Torres, Sidney B. Martínez-González, Alan D. Lozano-Luján, María C. Martínez-Vázquez, Perla Velasco-Elizondo, Idalia Garza-Veloz, Margarita L. Martinez-Fierro

**Affiliations:** Unidad Académica De Medicina Humana y Ciencias De La Salud, Universidad Autónoma De Zacatecas, Zacatecas, Mexico

**Keywords:** amniotic membrane, placenta, graft, surgery, regenerative medicine

## Abstract

The amniotic membrane (AM) is the inner part of the placenta. It has been used therapeutically for the last century. The biological proprieties of AM include immunomodulatory, anti-scarring, anti-microbial, pro or anti-angiogenic (surface dependent), and tissue growth promotion. Because of these, AM is a functional tissue for the treatment of different pathologies. The AM is today part of the treatment for various conditions such as wounds, ulcers, burns, adhesions, and skin injury, among others, with surgical resolution. This review focuses on the current surgical areas, including gynecology, plastic surgery, gastrointestinal, traumatology, neurosurgery, and ophthalmology, among others, that use AM as a therapeutic option to increase the success rate of surgical procedures. Currently there are articles describing the mechanisms of action of AM, some therapeutic implications and the use in surgeries of specific surgical areas, this prevents knowing the therapeutic response of AM when used in surgeries of different organs or tissues. Therefore, we described the use of AM in various surgical specialties along with the mechanisms of action, helping to improve the understanding of the therapeutic targets and achieving an adequate perspective of the surgical utility of AM with a particular emphasis on regenerative medicine.

## Introduction

**FIGURE 1:**
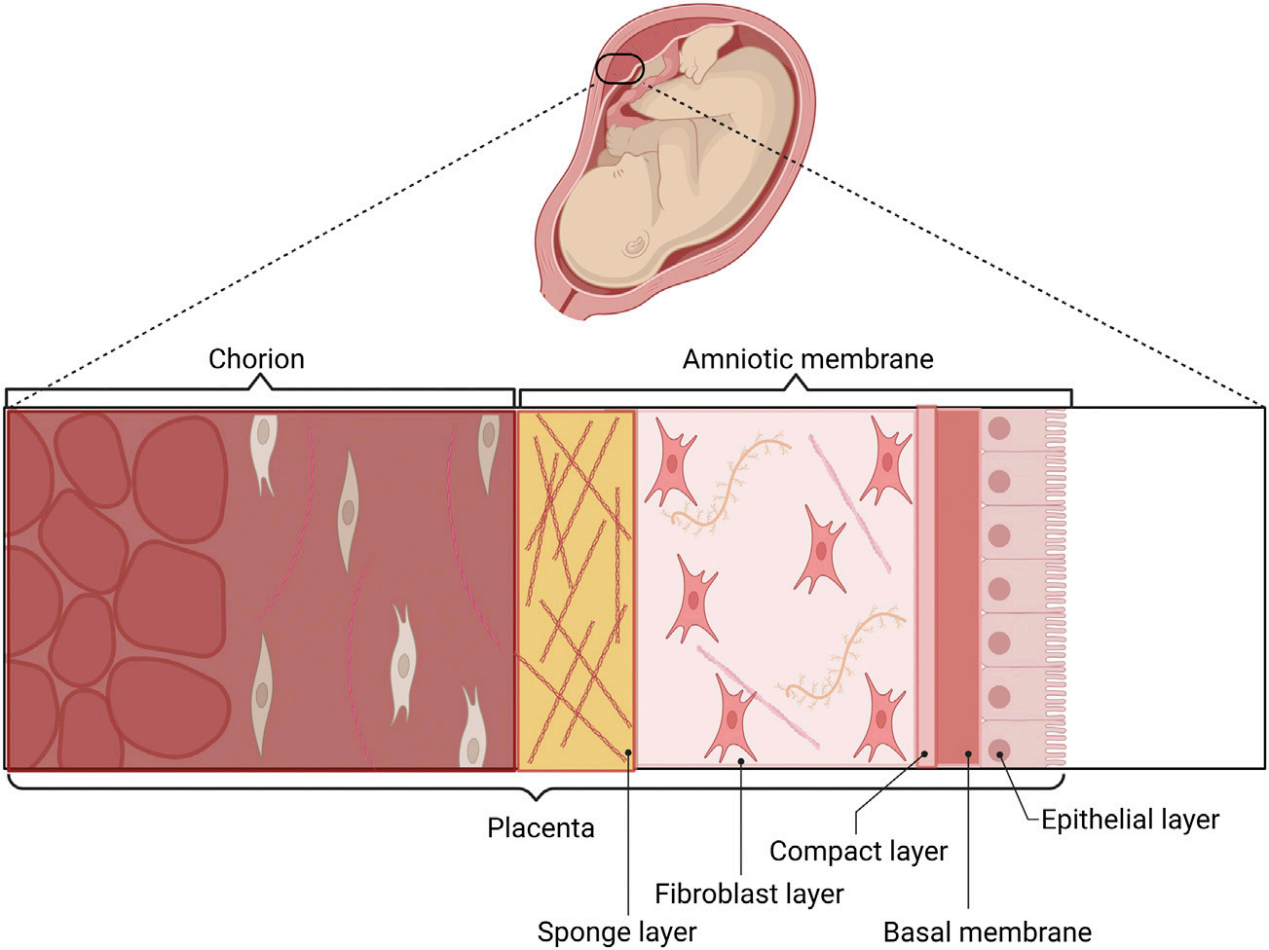
Components of the placenta and AM. The AM and the chorion are the two layers of the placenta. The epithelial layer, basal membrane, compact layer, fibroblast layer, and sponge layer are components of AM. The epithelial surface of AM is in con-tact with the fetus, and the chorionic layer is in contact with the maternal uterus.

The placenta is extra-embryonic tissue, generated during the gestation process. It has various functions: it protects the fetus from environmental injuries, regulates water, growth factors, cytokines, bioactive molecules, and minerals around the fetus, and plays essential roles during parturition (Toda et al., 2007; Mamede et al., 2012). The components are the chorion and amniotic membrane (AM) (Mamede et al., 2012). AM has different layers: epithelial cell layer, basement membrane, compact layer, fibroblast layer, and sponge layer (Figure 1) (Farhadihosseinabadi et al., 2018). The components of AM include pluripotent cells: human amniotic epithelial cells (hAECs) and human amniotic mesenchymal cells (hAMCs), nutrients, growth factors, extracellular matrix proteins, and cytokines. All of these make AM an excellent therapeutic tool, with regenerative, immunomodulatory, analgesic, anti-scarring, anti-microbial, pro or anti-angiogenic (surface dependent), and promotion of tissue growth properties (Okazaki et al., 1981; Bryant-Greenwood et al., 1987; Toda et al., 2007; Abbasi-Kangevari et al., 2019; Qiu et al., 2020).

The therapeutic applications of AM are diverse, and the mechanisms of action of each one are different. The immunomodulatory function is related to the suppression of pro-inflammatory cytokines such as tumor necrosis factor α (TNFα), interleukin 1 (IL-1), IL-6, and IL-8 by molecules such as interleukin-1 receptor antagonist (IL-1RA) and IL-10, by reduction of IRAK-4 expression, with consequent reduction of phosphorylation at the p65 subunit of NF-κB, and of the three MAPKs: JNK1/2, p38 and ERK1/2. (Fortunato et al., 1996; Fortunato et al., 1997; , 1998; Magatti et al., 2020). The anti-scarring effect results from fibroblast inhibition through transforming growth factor beta (TGF-β), which prevents myofibroblast differentiation (Grande, 1997; Jester et al., 1999). Some molecules such as α and β defensins, elafin, and leukocyte proteases act as a barriers against bacterial infiltration and confer antimicrobial functions (Mamede et al., 2012; Ramuta et al., 2021). The regulation of pathways like ERK-1/2-MAPK (mitogen-activated protein kinase) and some growth factors such as vascular endothelial growth factor A (VEGF-A), hepatocyte growth factor (HGF), and fibroblast growth factor 2 (FGF-2) can increase perfusion and capillary density. However, it depends on the surface AM on which is placed. It was observed that when AM was placed epithelial side up, the number of vessels and their lengths were increased, but when AM was placed mesenchymal side up, angiogenesis decreased (Kucukerdonmez et al., 2007; Kim et al., 2012; Niknejad et al., 2013; Farhadihosseinabadi et al., 2018). Growth factors, including epidermal growth factor receptor (EGF-R), insulin-like growth factor 2 (IGF-2), neurotrophin-4 (NT-4), macrophage colony-stimulating factor (M-CSF), granulocyte/macrophage colony-stimulating factor (GM-CSF), nutrients and components of the extracellular matrix in AM promote the growth of different tissues (Toda et al., 2007). In 1910, John Davis used AM for therapeutic purposes like skin grafts in some skin injuries (Davis, 1909); later, AM was used in various wounds like burns, diabetic foot ulcers, venous ulcers, ocular injuries, and uterine adherences (Kheirkhah et al., 2008; Zelen et al., 2013; Zheng et al., 2018; Shpichka et al., 2019). The surgical resolution or surgical treatment of some medical problems is an essential part of the treatment of many patients. In this article, we describe in detail the properties of AM (immunomodulatory, antiscarring, antimicrobial, angiogenic site-dependent and tissue growth promoting) and at the same time, we integrated the wide range of surgical uses of AM. On the other hand, we projected the possible medical areas or pathological processes in which MA has not yet been used and which could be a good therapeutic option.

## Properties of the amniotic membrane

### Immunomodulatory function

Immunomodulation is a process that modifies the response of the immune system by altering and interfering with its functions. This modification can generate suppression or stimulation of the immune system. The purpose of this interference in the immune response is to regulate and maintain a balance that favors a homeostatic course (Sehar et al., 2008). The immunomodulatory mechanisms involved work at different levels of the immune system. They can inhibit or stimulate specific leukocyte populations, function, proliferation, phenotype or the production of cytokines and growth factors that they may secrete (Bascones-Martinez et al., 2014). Inflammation is a process in which the immune system is largely involved and therefore a process that can be immunomodulated at different levels by different treatments as AM (Bulati et al., 2020). This is an adaptive biological response of vascularized tissues that is generated in response to a disease or injury event. It is characterized by different phases or stages different classes of inflammatory mediators, the pathways that control their production, and their mechanisms of action (Medzhitov, 2010). The presence of viruses, bacteria, fungi, parasites, trauma, toxins and necrotic cells are considered triggers of the inflammatory process. Initially, vascular changes are described, characterized by vasodilatation, changes in blood flow and increased permeability. The main generators of these changes are histamine and nitric oxide (Tommie, 2020). This generates the presence of exudate at the interstitial level that will be clinically reflected by the presence of edema, erythema, increased temperature and pain. At the endothelial level, adhesion molecules are expressed that favor the migration, adhesion and infiltration of cells of the innate immune system towards the lesion site. Once leukocytes are present at the site of injury, the release of inflammatory cytokines (IL-1,6, TNF-α, IFN-α), chemokines (CCL2, CXCL8) and other substances increases, amplifying the inflammatory process and favoring leukocyte activation and function in order to restore the homeostasis of the damaged tissue (Okin and Medzhitov, 2012; Bennett et al., 2018; Bentley and Little, 2021).

Different cytokines in the AM matrix are key to anti-inflammatory and analgesic function. The human amniochorionic membrane (hACM) was placed like an organ explant system and stimulated with lipopolysaccharide (LPS) in culture. It was evident that IL-10, present in hACM, downregulated IL-6, IL-8, and TNFα (Fortunato et al., 1996; Fortunato et al., 1997; Fortunato et al., 1998). In *in vitro* assays when hAMSCs are in the presence of PBMC activated by inflammatory stimuli, they begin to synthesize the soluble immunosuppressive factors IDO, PGE2 and IL-10. However, hAMSCs are not solely dependent on the secretion of soluble factors. It has been observed that inhibition of PBMC proliferation is also by cell-to-cell contact, accompanied by overexpression of PDL-1 in hAMSCs as well as PD-1 in activated PBMCs (Bulati et al., 2020). The signaling pathways involved in the anti-inflammatory process through hAMCs are the reduction of IRAK-4 expression, with the consequent reduction of phosphorylation in the p65 subunit of NF-κB, and of the three MAPKs: JNK1/2, p38 and ERK1/2(Magatti et al., 2020). RNA and DNA analysis of hAECs and hAMCs showed high expression of anti-inflammatory proteins such as IL-RA, all four tissue inhibitors of metalloproteinases (TIMPs), collagen XVIII, and IL-10 (Hao et al., 2000). Co-culture of corneal limbal epithelial cells with AM stroma stimulated with LPS showed that IL-1α and IL-1β transcripts and proteins were significantly reduced by AM stromal matrix compared with plastic culture, whether LPS was added or not (Solomon et al., 2001b). Stimulation *in vitro* of hAMCs with IFN-γ can induce PDL-1 expression, increased IDO production, and up-regulation of different miRNAs involved in the regulation of proteins that control the T cell activation/anergy pathway such as the monocyte differentiation pathway. These miRNAs interact with IGF1R, PI3K, GRK2, CDK6, RAS/MAPK, AP1, PRDM1, NUFIP2, PRNP, and IRF-4, genes that govern T-cell survival/proliferation, immune response, and anergy (Bulati et al., 2020). The interaction of amnion-derived cells (ADC) and natural killer (NK) lymphocytes generated inhibition of NK cytotoxicity and downregulated monocyte cytokine production. This was related to the secretion of IL-10 and prostaglandin E2 (PGE2) in the supernatant by ADC; this resulted in a downregulation of the expression of activated NK receptors and their production of IFN-γ. Moreover, monocytes secreted less TNF-α and IL-6 (Li et al., 2015; Tan et al., 2015). AM improved the phagocytosis of neutrophils, but at the same time, decreased their oxidative burst capacity and neutrophil extracellular traps (Navas et al., 2018; Alipour et al., 2020). Another immunomodulatory property of hAECs was observed through the trogocytosis process, where cell-to-cell transfer of human leukocyte antigen G (HLA-G) from hAECs to effector T cells was observed, which may account for the acquisition of a regulatory T cell (Treg) phenotype independent of FoxP3 transcription. Also, hAECs and hAMCs influenced the chemotaxis and polarization of macrophages from M1 to M2 switch and enhancing M2 macrophage features (Tan et al., 2015; Magatti et al., 2017; Ragni et al., 2021; Papait et al., 2022). In experimental autoimmune uveitis (EAU) and experimental autoimmune thyroiditis (EAT) in rats, hAEC inhibited the retinal and thyroid infiltration of macrophages and T cells by downregulating T helper (Th)17 cells (Th17) and upregulating Treg cells, as well as decreasing IL-17 and IFN-γ and increasing IL-10 and TGF-β levels (Li et al., 2018; Tan et al., 2018). On the other hand, low concentrations of placental NHERF1 (N+/H+ external exchanger regulatory factor 1) are related to low NF-κB activation, which generates low pro-inflammatory cytokine levels (Kammala et al., 2020). hAEC supernatant significantly limits the inflammatory process, decreasing the migration of neutrophils and macrophages and inhibiting macrophage inflammatory protein 2 (MIP-2), which reduces the proliferation of B and T lymphocytes (Li et al., 2005; Papait et al., 2022).

**FIGURE 2:**
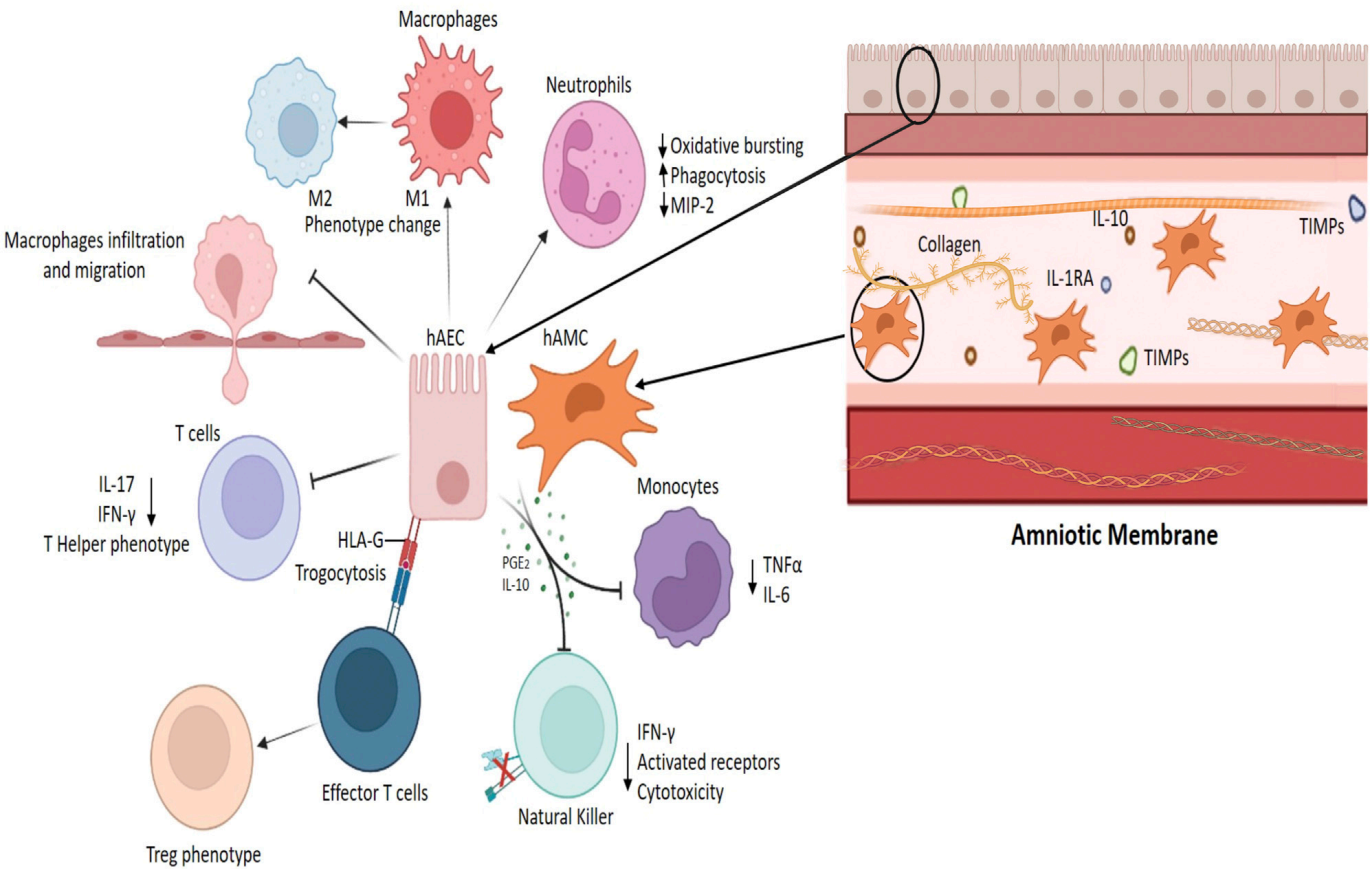
Immunomodulatory function of the amniotic membrane. Amnion-derived cells (hAEC, human amniotic epithelial cells; hAMC, human amniotic mesenchymal cells) reduce the secretion of inflammatory cytokines (TNFα, IFNγ, IL-6, and 17) by monocytes and lymphocytes, favor the generation of Treg lymphocytes (trogocytosis process), induce the phenotypic switch of inflammatory macrophages M1 to M2, and inhibit macrophage migration and infiltration into tissues (Li et al., 2015; Tan et al., 2015).

It is important to note that AM has the ability to generate immunomodulation of the inflammatory process and as a result limit and decrease the clinical symptomatology of the inflammatory process, the generation of analgesia, decrease of interstitial edema and restoration of normal vascular status are the result of immunomodulation at different levels: suppression of inflammatory cytokines, decrease of oxidative burst, limitation of chemotaxis, improvement of phagocytosis, change of cell phenotype and regulation of different cell types (Tan et al., 2015; Li et al., 2018). These are the mechanisms through which the cells present in AM (hAEC and hAMC) generate immunomodulation by targeting different cells of the immune system (see Figure 2).

### Anti-scarring function

Wound healing is a complex process, characterized by the spatial and temporal effect of various cell types with different functions but which interact in the different phases of healing: hemostasis, inflammation, growth, re-epithelialization, and remodeling. Changes in the microenvironment, mechanical forces, oxygen levels, cytokines, chemokines, extracellular matrix, and growth factors present at the site of injury directly impact the process. If there is an imbalance of these factors, healing is impaired, as occurs in chronic wounds, hypertrophic scars and keloid formation, where the fibrosis process contributes to a great extent to abnormal wound closure (Gurtner et al., 2008; Rodrigues et al., 2019). Fibrosis is not a disease, but rather an outcome of the tissue repair response (Henderson et al., 2020). Fibrotic tissue is defined by the excessive accumulation of extracellular matrix (ECM) components such as collagen and fibronectin, causing the disruption of normal tissue architecture and function (Eming et al., 2014). Different cytokines like IL-1, IL-6, IL-11, IL-13, IL-17, TNFα, and TGF-β have a major role, together with cells of the immune system (macrophages, monocytes, and neutrophils), as well as mesenchymal stromal cells, fibroblasts, and myofibroblasts in fibrosis development (Henderson et al., 2020). Myofibroblasts can produce excessive amounts of ECM and exert tractional forces across the ECM, resulting in the distortion of tissue architecture (Wynn and Ramalingam, 2012). Macrophage-derived amphiregulin, an epidermal growth factor receptor ligand, can induce the differentiation of mesenchymal stromal cells into myofibroblasts *via* integrin αv-mediated activation of TGF-β. Also, macrophages crosstalk with contractile fibroblasts to generate deformation fields the fibrillar collagen matrix (Minutti et al., 2019; Pakshir et al., 2019). TGF-β can overexpress ECM components such as collagens, proteoglycans, and metalloprotease inhibitors. Conversely, it was decrease the expression of metalloproteases (MMP) (Lawrence, 1996; Grande, 1997; Jester et al., 1999). Conjunctival, limbal, and pterygial fibroblasts cultured on the stromal matrix of AM showed suppression of transcripts of TGF-β 1, 2, and 3 and TGF-βR I, II, and III. At the same time, AM can suppress downstream proteins in the TGF-β signaling pathway such as alpha smooth muscle actin (αSMA), integrin β1, α5, CD44, fibroblast growth factor receptor 1 (FGF-R1/flg), and fibronectin. (Tseng et al., 1999; Lee et al., 2000). TGF-β1 and CD44 are described as a part of the mechanism by which the fibroblast to myofibroblast differentiation is mediated (Midgley et al., 2013). Fibroblasts cultured and differentiated into myofibroblasts and then re-seeded onto a cryopreserved amniotic membrane stromal surface, underwent a reversion to the fibroblast phenotype (Li et al., 2008). Specifically, heavy chain-hyaluronic acid/pentraxin 3 (HC-HA/PTX3) is a component purified from AM. When added to cultured human corneal fibroblasts and myofibroblasts, it was shown to suppress canonical TGF-β1 signaling and led to phenotypic reversal to keratocan-expressing keratocytes through the activation of bone morphogenetic protein (BMP) signaling (Zhu et al., 2020). In addition, keratocytes isolated from central corneal buttons and cultured on the stromal matrix of human AM maintained their characteristic morphology and keratocan expression and were prevented from acquiring a fibroblast morphology or expressing fibrotic proteins (Espana et al., 2003). The development of the fibrotic process is difficult to suppress because the formation mechanism is multifactorial. Using a single substance to try to inhibit scar formation is often insufficient. AM can suppress the pathological generation of fibrotic tissue at different levels, and can result in the reduction or absence of scarring and the preservation of tissue architecture and functionality.

### Antimicrobial function

It is estimated that about 24% of patients are affected by healthcare-associated sepsis (HAS) and 52.3% of these patients treated in an intensive care unit die each year. Deaths are increased two to threefold when infections are antimicrobial resistant (AMR) (Organization, 2022). The UK Government argued that AMR could kill 10 million people per year by 2050. The principal six pathogens contributing to the burden of AMR in 2019 were *E. coli,* *S. aureus, K. pneumoniae, S. pneumoniae, A. baumannii,* and *P. aeruginosa*, and are considered to be priority pathogens by the WHO (Antimicrobial Resistance, 2022). The mechanisms of antibiotic resistance include modifications of the antibiotic molecule (Wilson, 2014), decreased antibiotic penetration and efflux (Pages et al., 2008), changes in target sites, and resistance due to global cell adaptations (Bayer et al., 2013; Aldred et al., 2014). A homogenate of AM had anti-bacterial activity against 7 out of 11 tested multidrug-resistant strains; the greatest effect was on methicillin-resistant *Staphylococcus aureus* (MRSA). It was evaluated in a normal microenvironment and cancerous urinary bladder urothelia, where AM did not affect the viability, number, and ultrastructure of urothelial cells (Ramuta et al., 2021), but had a bacteriostatic effect on uropathogenic *Escherichia coli* (UPEC) and *S. aureus* (Sket et al., 2019; Ramuta et al., 2020). Chorion suspended in agar and liquid cultures showed a marginal inhibitory effect, but the most pronounced inhibition was obtained for *Streptococcus* group A, *S. aureus,* and *S. saprophyticus* by hACM (Kjaergaard et al., 2001). Human cryopreserved viable amniotic membrane (hCVAM) on wounds promoted closure and reduced wound-related infections in treating chronic diabetic foot ulcers compared with the standard of care. *In vitro*, AM demonstrated a significant reduction of ESKAPE bacteria (*Enterococcus faecium, Staphylococcus aureus, Klebsiella pneumoniae, Acinetobacter baumannii, Pseudomonas aeruginosa or Enterobacter aerogenes*) (Mao et al., 2016). AM extract and chorionic membrane extract (AME/CME) inhibited *S. pneumoniae* growth (Yadav et al., 2017), oral streptococci (Palanker et al., 2019), and *P. aeruginosa, S. aureus* and methicillin-resistant *S. aureus* (Mao et al., 2017).

The AM effect involves different molecules; some of them are α and β defensins, which possess anti-bacterial, anti-viral, and anti-fungal activity, particularly defensin β3, which possess a significant anti-bacterial effect. In addition, the expression of defensins increases when the LPS of bacteria is present (Buhimschi et al., 2004; Szukiewicz et al., 2016; Mao et al., 2017). Acidic peptides such as leukocyte protease secretion inhibitor and elafin possess anti-microbial and anti-protease properties. Cathelicidin LL-37 is an innate anti-microbial polypeptide secreted by hAMCs (Tehrani et al., 2017; Farhadihosseinabadi et al., 2018; Ramuta et al., 2021). The presence of immunoglobulins, especially IgA, is one of the main anti-microbial components. On the other hand, Fas ligand on the cell surface and the soluble form mediates apoptotic functions, which is important for anti-microbial control (Zare-Bidaki et al., 2017; Qiu et al., 2020). These properties are currently considered important because AM components have been shown to inhibit the growth of multidrug-resistant bacteria, so AM has been considered a therapeutic alternative in the treatment of these microorganisms (Ramuta et al., 2021). The search for new solutions to anti-microbial resistance continues to be a priority. It is a problem that has not been solved yet. Anti-microbial resistance continues to grow, and the generation of new anti-microbials is limited. This generates the search for alternative solutions for this problem. The anti-microbial functions of the AM make it a therapeutic alternative to solve this condition.

### Site-dependent angiogenic function

In the early phase of wound healing and repair, numerous new capillaries appear in the neostroma; it is called granulation tissue and begins to form approximately 4 days after injury. Macrophages, fibroblasts, and blood vessels move into the wound space during tissue repair (Heimark et al., 1986). New blood vessel formation (angiogenesis) is a critical component of wound healing. These events require a dynamic, temporally and spatially regulated interaction between endothelial cells, angiogenic factors, and surrounding ECM proteins (Cheresh et al., 1989; Tonnesen et al., 2000). When evaluating the angio-vasculogenic properties of hAMCs by determining their therapeutic effects in experimental ischemia, significantly higher levels of pro-angiogenic genes were observed, particularly VEGF-A, angiopoietin-1 (ANG-1), HGF, and FGF-2, compared to adipose-derived mesenchymal stem cells (ADMSC). In another study, implantation of hAMCs augmented blood perfusion and capillary density in an ischemic hindlimb model (Kim et al., 2012). Additionally, hAMCs transplanted into injured sciatic nerves augmented blood perfusion and increased intraneural vascularity (Li et al., 2014). Moreover, hAMC conditioned medium (CM)/human umbilical vein endothelial cell (HUVEC) coculture demonstrated angiogenic capacity *in vivo* and *in vitro*; as part of this process, MMPs showed a proteolytic role, as MMP2 and MMP9 were increased on the protein level in hAMSCs in 3D culture conditions (Jiang et al., 2015). Regarding the RNA level, the circular RNA 100290 (circ-100290) was found to act *via* miR-449a, enhancing the expression of endothelial nitric oxide synthase (eNOS) and VEGF-A, and increased expression of circular RNA ATP binding cassette subfamily B member 10 (circ-ABCB10) upregulated levels of VEGF-A, explaining the pro-angiogenic role of hAMCs-CM on HUVEC (Tang et al., 2020a; Tang et al., 2020b). Proteomic and microarray evaluations of hAMCs-CM/HUVEC elucidated potential signaling pathways through which tissue-derived factors induce pro-angiogenetic phenotypes. *In vivo*, the addition of CM resulted in increased CD31 and α-SMA; *in vitro*, CM resulted in significant increases in endothelial proliferation, migration, and the expression of GM-CSF, HGF, and TGF-β3, all of them mediated by ERK1/2 pathway signaling (McQuilling et al., 2019a). This angiogenic function is essential in a variety of standard and pathological processes. Physiologic angiogenesis is a critical factor in wound and fracture healing, endometrial growth, embryonic implantation, and formation of the placenta. But, it also takes place in the pathophysiology of tumor growth, metastasis, rheumatoid arthritis, retinopathies, and others (Folkman, 1995). The use of AM at the corneal level showed anti-angiogenic capacity. When it was used in post-pterygium surgeries, there was a decrease in the vascularization process where the fibrous pterygium lesion was located (Kim and Tseng, 1995; Jiang et al., 2006; Kucukerdonmez et al., 2007). AM contains some proteins that inhibit new blood vessel formation, such as collagen IV, laminin, and integrins 4 and 6, which have been related to the suppression of angiogenesis at the corneal level (Kim and Tseng, 1995). Endostatin, thrombospondin 1, T cell immunoglobulin mucin 1, 2 (TIM1,2), IL-1βA, IL-10, collagen XVIII, and pigment epithelial-dependent factor (PEDF) can prevent the migration of endothelial cells, inhibit their replication, or decrease the mobility of angiogenic factors. In this way, AM may inhibit the formation of new blood vessels (Liu et al., 2010; Hossain et al., 2019). Nevertheless, this function is AM surface-dependent. It has been observed that when AM was placed epithelial side up, the number of vessels and their lengths were increased, but when AM was placed mesenchymal side up, angiogenesis decreased (Niknejad et al., 2013).

### Tissue regeneration

Tissue repair is a universal phenomenon of multicellular organisms; when an injury occurs, the tissues involve a complex interplay between many cellular players such as keratinocytes, fibroblasts, endothelial cells of vessels, and recruited immune cells, and their associated extracellular matrix (Dekoninck and Blanpain, 2019). The restoration process is highly efficient, but when the damage is extensive, the repair process is abnormal and results in scar formation or substantial loss of original tissue structure and function (Martin and Nunan, 2015). AM can support tissue repair with three main elements: stem cells (hAECs and hAMCs), which retain the capacity to renew themselves and may be able to restore damaged tissue with high proliferation and differentiation; the scaffolds that support them; and growth and differentiation factors (Toda et al., 2007). The factors secreted by hAMCs (secretome) evaluated *in vitro* describe the secretome as cell-free therapy, where around 60 cytokines/chemokines have been found to be involved in chemotaxis, homeostasis of inflammatory cells and positive remodeling of the extracellular matrix, 200 growth factors and 754 miRNAs in extracellular vesicles (EV). Most of these miRNAs were related to the protection of tenocytes and chondrocytes, capable of improving musculoskeletal conditions (Ragni et al., 2020; Ragni et al., 2021). hAECs possess stem-cell-like plasticity, immune privilege, and paracrine properties. hAESCs have the potential to differentiate into all three germ layers under the proper conditions (Miki et al., 2005; Ilancheran et al., 2007). The hAMCs are derived from AM and amniotic fluid (AF). They are an excellent candidate in regenerative medicine compared with other mesenchymal stem cell (MSC) sources because of the ease of their acquisition, reduced donor damage, multipotency, low immune response, and acceptable ethical issue (Toda et al., 2007; Kim et al., 2014). As the scaffold, collagens, elastin, and other ECM components play an essential role in its biomechanical properties. The basal membrane allows better cell proliferation and differentiation and improves uniformity of cell outgrowth (Koizumi et al., 2000; Koizumi et al., 2007). Secondly, AM has a lot of growth factors and cytokines. hAMCs produce and secrete EGF-R, IGF-2, insulin-like growth factor binding proteins 2, 3, and 6 (IGFBP-2, 3, 6), and NT-4. Meanwhile, hAECs produce and secrete M-CSF, granulocyte colony-stimulating factor receptor (M-CSF-R), platelet-derived growth factor AB (PDGF-AB), placental growth factor (PLGF), and granulocyte colony-stimulating factor (G-CSF) (Grzywocz et al., 2014). All of them are present in AM and play an essential role in different functions such as cell activation, proliferation, or cell differentiation at other sites of the injury (Rashedul Islam et al., 2015). In the regenerative skin process, these molecules are relevant because some of them participate in the new tissue formation. This involves proteins such as GM-CSF, PDGFs, FGFs, VEGFs, and MMPs (Barrientos et al., 2014). In addition, some conditions such as older donors, higher gestational age, and the use of gamma rays as a sterilization mechanism can affect the concentrations of cytokines and growth factors. In the first two cases, the concentrations of FGF-2, HGF, KGF, NGF, and TGF-β1 are affected (Lopez-Valladares et al., 2010). Gamma rays can affect cells and amino acids such as tyrosine. It generates structural and functional changes in the proteins and has effects on AM functionality (Paolin et al., 2016).

## Obtaining and presentations of amniotic membrane for medical applications

The obtaining and presentations of AM for therapeutic are variated and it will depend of the medical application, the tissue availability and storage capacity (temperature). The AM procurement process consists of obtaining and separating the amnion from the umbilical cord and the chorionic portion by dissection. After a series of washes the umbilical cord and chorionic portion are discarded. The processing of the AM in the laboratory should be carried out with an aseptic technique using sterile reagents and instruments in a laminar flow cabinet. Later AM is segmented into portions of the required size, incubated with Dulbecco’s Modified Eagle Medium containing antibiotic cocktail (DMEM), and spread out on nitrocellulose paper for later storage in sterile bags. To preserve viable cells, AM should not be sterilized by radiation or any other method. The asepsis of the process is confirmed by USP sterility tests (Duan-Arnold et al., 2015; Dhall et al., 2018). The most frequent form in which AM is used is fresh (when it is used within the first hours after its processing and separation from the rest of the placenta). AM cryopreservation is carried out by adding dimethyl sulfoxide (DMSO) (Duan-Arnold et al., 2015) and 1% human serum albumin in saline solution (Dhall et al., 2018) or DMEM and glycerol at the ratio of 1:1 (Lee and Tseng, 1997; Sharma et al., 2018). The freezing process is controlled and progressive until reaching -80°C, which allows the preservation of proteins, growth factors, and cell structures for a long time. Lyophilization or dry freezing of AM consists in the removal of water from the tissue, allowing the AM and its tissue components to be stored at room temperature or frozen for long periods of time, respectively. Air drying is a cheap and easy technique used to remove moisture from the membrane, facilitating its storage at room temperature, however it needs sterilization through gamma rays for later use (von Versen-Hoeynck et al., 2008; Rodriguez-Ares et al., 2009; Rodriguez-Ares et al., 2009). However, the loss of water from the tissue could modify the state of cellular components and modify AM functionality. Finally, de-epithelialization of AM by physical or chemical methods removes the cellular components of the membrane, limiting the generation of a rejection response to the maximum; however these procedures also can damage the state of the AM (Riau et al., 2010; Fenelon et al., 2021).

## Surgical applications of the amniotic membrane

**FIGURE 3:**
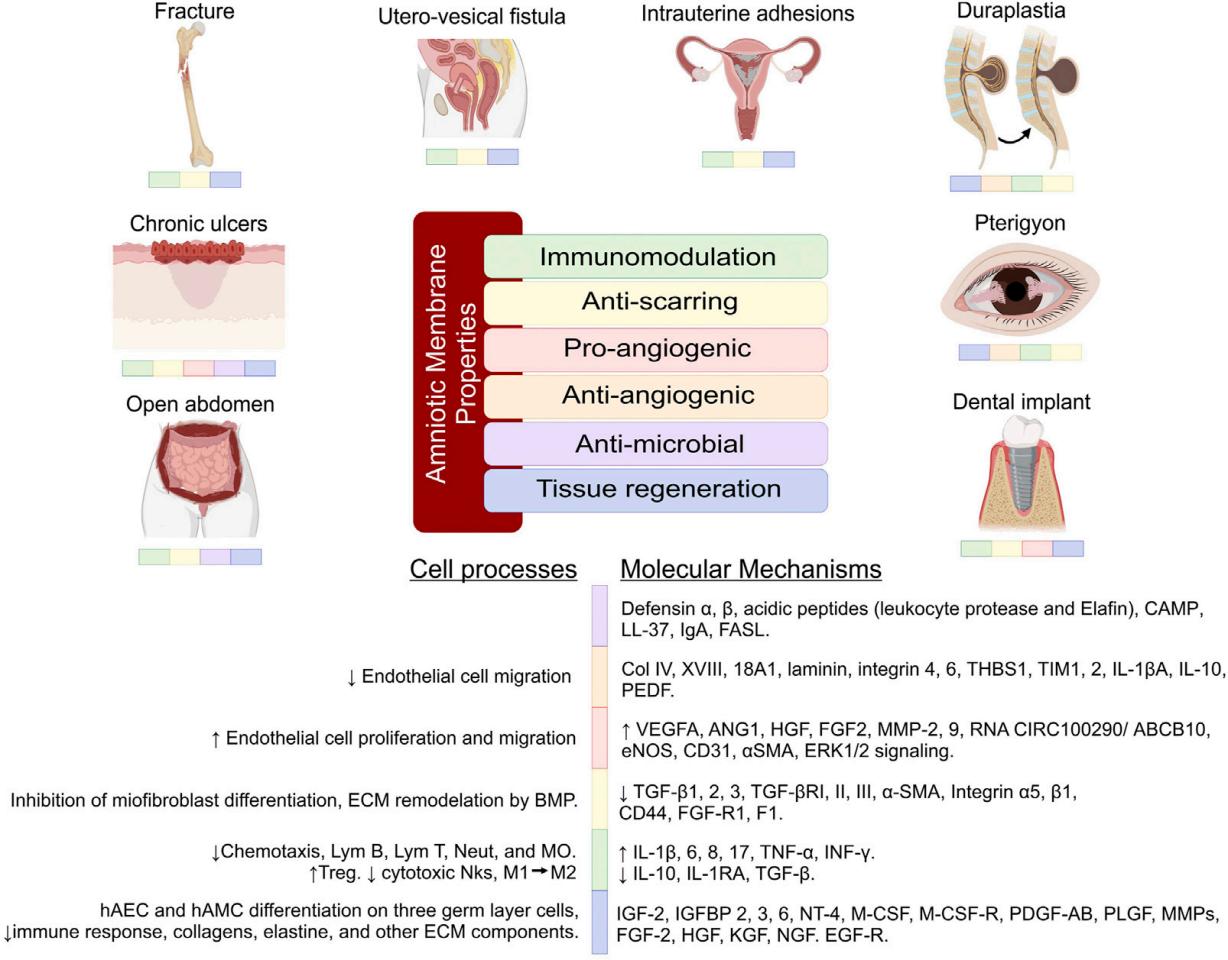
Therapeutic effects of the amniotic membrane and the molecular mechanisms. Each disease has a specific pathophysiology; therefore, the therapeutic effects of AM are different for each of the pathologies. The molecular mechanisms involved are varied, but not all of them are involved in the resolution of the diseases. The figure reinforces the knowledge of the therapeutic effects of AM and also helps to understand future applications of this tissue. Lym, Lymphocyte; MØ, Macrophages; Neut, Neutrophils; MO, Monocytes; Treg, Lymphocyte regulator; NKs, Lymphocytes Natural Killers; CD, Cluster differentiation; αSMA, Smooth muscle actin; ERK, Extracellular signal-regulated kinase; Col, Collagen; FGF-R, Fibroblast growth factor receptor; ECM, Extra cellular matrix; MMP, Matrix metalloproteinases; BMP, Bone morphogenetic protein; THBS1, Thrombospondin 1; TIM, T cell immunoglobulin mucin; IL, Interleukin; VEGFA, Vascular endothelial growth factor A; ANG1, Angiopoietin 1; HGF, Hepatocyte Growth Factor; EGF, Epidermal growth factor; FGF, Fibroblast growth factors; eNOS, Endothelial nitric oxide synthase; PDEF, Pigment Epithelium-Derived Factor; PDGF, Platelet-derived growth factor; PLGF, Placental Growth Factor; KGF, Keratinocyte growth factor; NGF, Nerve growth factor; TGF, Transforming growth factor; TNF, Tumor necrosis factor; INF, Interferon; IGF, Insulin-like growth factor; IGFBP, Insulin-like growth factor-binding protein; NT, Neurotrophin; M-CSF, Macrophage-colony stimulating factor.

Each human disease has a specific pathophysiology and therefore the altered molecular mechanisms involved are varied. The wide range of biological properties of AM (immunomodulation, anti-scarring, pro-angiogenic/anti-angiogenic, antimicrobial and tissue regeneration, among others), provide a wide variety of possibilities for successful applications and clinical procedures (Figure 3). However, because the differences in the etiology of each disease not all of the biological properties of AM will be involved in its resolution, therefore the therapeutic effects of AM are different for each of the pathologies. In the following paragraphs we will describe different surgical procedures and the presentation or form of application of AM in different surgical areas. At the same time, the clinical results obtained after its therapeutic use are described simultaneously, integrating the molecular changes of components involved in the pathophysiology of diseases undergoing surgical treatment after the use of AM.

### Gynecology

**FIGURE 4:**
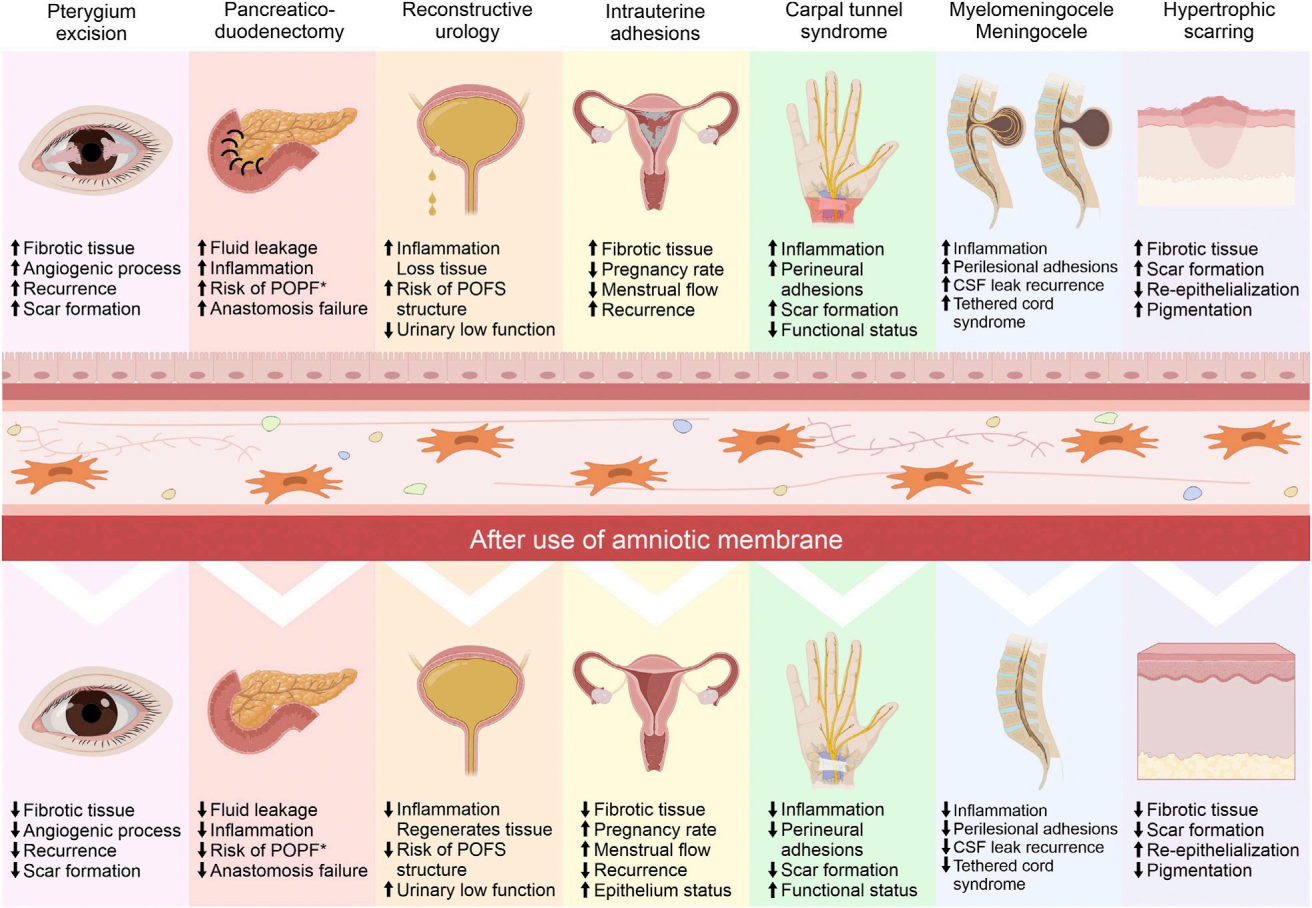
Impact of the amniotic membrane (AM) in different surgical procedures. The use of AM in surgeries in different surgical areas is used for different biological purposes, where the modification of pathophysiological processes impacts on the functionality, state and tissue condition of different organs, all of which is reflected in the postoperative clinical results. POFS, Post-operative fistulation and stenosis; POPF, Postoperative pancreatic fistulation; CSF, Cerebrospinal fluid.

Within gynecology, the AM has been used to treat different etiologies and for different purposes. Epithelial regeneration in vaginal reconstruction surgeries has been one of the objectives since the beginning of the last century (A., 1934). Vaginal agenesis patients (Mayer-Rokitansky-Kuster-Hauser syndrome) undergo vaginoplasty surgery as a reconstructive treatment. The utility of AM in this procedure has been demonstrated by biopsy showing epithelialization of normal depth and caliber 8 weeks after surgery, without exudates, scars, dryness, or adhesions; no side effects were reported up to 2 years after surgery of the new vaginal cavity. The vaginal size achieved was between 8 and 10 cm, and at the same time, an average score of 30–32 points was achieved on the female sexual function index (FSFI) (Tancer et al., 1979; Hughes and Spence, 1988; Nisolle and Donnez, 1992; Avsar et al., 2016; Vatsa et al., 2017). In vaginal ulcers caused by vaginal mesh, treatment with cryopreserved AM in patients with lesions 5–25 mm in size was reported 27 months later; only one patient presented ulceration of less than 3 mm, and before surgery two of the seven patients presented dyspareunia, as well as after. With AM treatment, no patient reported this condition, and during this time no adverse effects were reported (Lau et al., 2020). In an attempt to avoid alterations in the healing process, frozen and fresh AM has been used preventively and therapeutically in the repair of utero-vesical fistulas; 7 days after surgery, cystography showed no urinary leakage and no evidence of tissue rejection (Barski et al., 2015; Tahereh Poordast and Doustfatemeh, 2019). In some pathologies, scarring and fibrosis are exaggerated, it produces intrauterine adhesions or intrauterine synechiae after surgical procedures. Clinical trials have compared the use of AM with chitosan showing a recurrence rate of adhesions in the first month with the use of chitosan of 47.9%, while with AM this was 15.4%. Complications were evaluated 3 months after surgery and it was shown a recurrence rate of 37.5% and 3.8%, respectively. At the same time, an increase in endometrial thickness was reported in the AM treatment group compared to the group treated with chitosan (Li C. et al., 2020). Other works have demonstrated a decrease in the recurrence of adhesions at 4 and 12 weeks after treatment with fresh and dried AM through hysteroscopy using the uterine adhesion index. They reported improvements in menstrual flow 3 months after treatment in 85%, while this was 66.3% in the control group (Amer and Abd-El-Maeboud, 2006; Gan et al., 2017; Peng et al., 2017). The pregnancy rate was reported to be up 80% with fresh AM (Amer et al., 2010; Chatterjee et al., 2020). It has been reported that improvements in endometrial tissue can be demonstrated by histology and immunohistochemistry (Amera et al., 2012). Figures 3, 4 shows the impact of the AM in different surgical procedures, including gynecology (intrauterine adhesions) showing the modification of pathophysiological processes and molecular mechanisms, impacts on the functionality, state and tissue condition, which is reflected in the postoperative clinical results.

### Plastic surgery

This surgical area focus on improving functionality, esthetics, and reducing the sequelae or disabilities that may result from birth defects, injury, disease, or aging (2022). AM has been used as a micrograft in microsurgeries performed in the abdomen, chest, upper and lower limbs in patients with granulation lesions due to burns, trauma, and venous ulcers, showing complete epithelialization of the lesion 7–10 days after the procedure (M, 1995). In toxic epidermal necrolysis patients have been compared in a lyophilized form with synthetic skin substitutes. It has been shown that AM produces re-epithelialization within the first 48 h. The wound healed 3 weeks later, and complete recovery of the skin without pigmentation or fibrosis was observed 3 months later, while synthetic skin substitutes showed slower recovery and pigmentation in the lesion area (Lipovy et al., 2021). In similar cases, others described the re-epithelialization of 90% of the total body surface area (TBS) affected by toxic epidermal necrolysis (Lyell’s syndrome) 14 days after the application of fresh and cryopreserved AM and 100% of the TBS at day 24. In addition to this, the regenerative and immunomodulatory capacity was histologically demonstrated (B. Azzena et al., 2018). Similarly, in adult patients with Stevens-Johnson syndrome (Klama-Baryla et al., 2017; Klama-Baryla et al., 2020), AM has been used as a treatment for the drop of hypertrophic scarring after burns, where it led to a 64% reduction in this type of scarring (Mohammadi et al., 2017). In this process, it has been observed that AM decreases TGF- β1 and α-SMA in dermal myofibroblasts, resulting in the modification of the extracellular matrix components and thus in the reduction of hypertrophic fibrosis (Moreno et al., 2021). It has analgesic functions, and favors early closure and healing of chronic wounds secondary to burns in different body regions in both children and adults (Mostaque and Rahman, 2011; Mohammadi et al., 2013a; Mohammadi et al., 2013b). In surgeries of cleft palate repair (palatoplasties), cryopreserved AM was used to achieve adequate oral intake 5 days after surgery, without bleeding during surgery, without fever, allergic reactions, wound dehiscence, infections, and adequate epithelialization of wounds (Martelloni et al., 2019; Fujiwara et al., 2022). AM has been used as a preventive treatment for tendon adhesion after repair. It has immunomodulatory characteristics that reduce the inflammatory process generated by these types of lesions and procedures. Part of the mechanism is explained by the limited secretion of IL-1β, IL-8, TNF-α, and TGF-β1 (Figure 3). When the inflammatory process is reduced, the density of tenocytes increases, the ECM is organized, and fibrosis is limited; all of these favor tendon repair (Hortensius et al., 2016; Hortensius et al., 2018; McQuilling et al., 2019b; McQuilling et al., 2019c; Kimmerling et al., 2019; Liu et al., 2019).

### Gastrointestinal surgery

The gastrointestinal system can suffer injuries that result in chronic diseases, such as cirrhosis and fibrosis. These problems have systemic repercussions and today are a health problem with an increasing incidence and a high percentage of morbidity and mortality (Blachier et al., 2013; Asrani et al., 2019); for this reason, it is urgent to find effective therapies for these pathologies. These diseases are progressive and reach the point where liver transplantation is the only option. In a rat model of cirrhosis generated by bile duct ligation, the fibrosis process was generated 2 weeks later. Fresh AM was placed on the hepatic surface totally or partially and, 4 weeks later, a decrease in the severity of fibrosis was histologically verified, due to a decrease in the amount of collagen deposits (Sant'Anna et al., 2011; Ricci et al., 2013; Mamede and Sant’anna, 2019). In post-operative pancreatic fistula after pancreatic resection, cryopreserved AM was applied with the aim of tissue regeneration and preventing fluid leakage at many surgical sites (Figure 4). It reduced inflammation, and preserved the integrity of anastomoses (Frigerio et al., 2019). It favored gastrointestinal tract re-epithelialization in duodenal lesion surgeries with high post-operative complications (Schimidt et al., 2010). Moreover, AM possesses anti-microbial, anti-viral, and anti-fungal characteristics that allow it to be used as a Bogota bag in patients with open abdomen (Tekin et al., 2008). In colonic surgeries, AM provides a beneficial effect. It has shown a delaying effect on intraperitoneal sepsis and provided a safer and stronger anastomosis. Histologically, AM treatment led to neoangiogenesis, fibroblast activity, collagen deposition, and hydroxyproline concentrations at significantly higher levels than in groups without AM (Uludag et al., 2009a; b). Similarly, acellular AM covering a sleeve gastrectomy cut surface area resulted in lower levels of PMNs in the injury; moreover, granulation tissue, vascularization, and fibroblastic proliferation were higher than the control, and while the presence of tissue edema was lower (Orman et al., 2020; Trejo et al., 2020). Also, cryopreserved AM has been used as a graft in the esophagus. It has shown interesting temporal results in macroscopic morphology, suggesting complete re-epithelialization in 90% of cases (Barret et al., 2014). Finally, cryopreserved AM has been used for the treatment of cryptoglandular anal fistula, where its use did not generate complications or the presence of pain or perilesional inflammation during the trans-operative period or in the check-ups carried out 24 h, 1 week, 1 month, and 3 months after surgery (Ratto et al., 2022).

### Traumatology

The different properties of AM have favored the repair of osteomuscular injuries in the area of traumatology in conditions such as carpal tunnel release surgery (CTRS) using frozen AM as a treatment for carpal tunnel syndrome, where the function of inhibiting inflammation, fibrosis, and favoring nerve repair is obtained by adhering AM in the median nerve, which in turn can improve clinical symptomatology compared to CTRS alone (Buentello-Volante et al., 2020). It also has anti-adhesion capacity in tendon repair, for example in the flexor tendon in zone II, as well as promoting tendon healing (Demirkan et al., 2002; Liu et al., 2019). The use of cryopreserved AM focused on bone tissue has demonstrated various benefits such as in fracture healing (Sari et al., 2019). The amniotic stem cells is used as part of an osteoinductive biomaterial for bone regeneration, either from the cellular components it contains (MSC) (Li J. et al., 2020) or from its acellular features (acellular natural ECM material) (Tang et al., 2018). When bone defects occur, and soft tissue infiltration into the defect space prevents neovascularization, this leads to pseudoarthrosis of a fracture; protected bone regeneration then occurs by the juxtaposition of a tissue-engineered bone graft (TEBG) with cellular or acellular elements of the AM in such a way that maintains the defect space, conducive to neovasculogenesis and infiltration of host osteogenic cells, leading to ultimate healing (Zhang et al., 2012; Tang et al., 2018). Also, in orthopedic surgery, it has been evidenced that AM-derived tissues are safe and non-tumorigenic, even producing a large number of growth factors that have shown promise as tissue scaffolds and as an aid in soft tissue regeneration. For example, a study of 14 patients undergoing foot and ankle surgery with tendon bandaging reported clinical improvement with reduced pain and more remarkable functional outcomes postoperatively compared to preoperative measurements (Heckmann et al., 2016).

### Neurosurgery

Surgical treatment of the nervous system limits the use of materials and tools because any damage to the neural tissue, however minimal it may seem, is avoided due to sequelae or repercussions. These injuries can generate irreparable harm to neural tissue. The fresh AM has been used as a substitute for the dura mater in reparative surgeries of myelomeningocele lesions ((Figure 4), where it has allowed for hermetic closure of the lesion and prevented cerebrospinal fluid (CSF) leakage, even in extensive muscle-fascial defects (Hasegawa et al., 2004; de Weerd et al., 2013; Marton et al., 2018; Schoellhammer et al., 2018; de Weerd et al., 2020; Chen. J et al., 2021). In skull base surgeries, dried AM was used as a patch graft for dural repair and to prevent CSF leakage. Two weeks after implantation, thick connective tissue completely enclosed the dried AM site. At 3 and 6 months after implantation, histological examination revealed the disappearance of the AM and the formation of membranous tissue (Tomita et al., 2012), as well as on the encephalic surface after decompressive craniectomies. The majority (96%) of AM grafts were integrated into the native dura. Histopathological analysis showed that AM had thick plates of dense fibrous tissue with small reactive vessels, reactive fibroblasts, and infiltrating lymphocytes (Marton et al., 2021). The fibrosis process and the generation of adhesions in the spine after epidural laminectomy have been reduced by the implantation of cryopreserved AM in the surgical site of animal model (Choi et al., 2011). In order to improve the nerve regeneration process, cryopreserved AM has been used in an animal model after performing neurorrhaphy, showing a decrease in fibrosis and perineural adhesions (Kim et al., 2010). Similarly, the repair of the common peroneal nerve in an animal model using cryopreserved AM improves the electrophysiological conditions and histological characteristics (Henry et al., 2009).

### Ophthalmology

The treatment of ophthalmologic conditions has widely integrated the use of AM. Ocular surface lesions generated by burns (chemical and thermal) have been treated with cryopreserved AM (Shimazaki et al., 1997). Reconstruction of this type of lesions with AM is often successful, and specular microscopy showed standard arrays, barrier function was almost completely recovered, and corrected visual acuity improved markedly, with minimal to mild scarring and no rejection response to the amniotic tissue (Shimazaki et al., 1997; Arora et al., 2005). In children, the use of AM has also shown to be an excellent therapeutic alternative for ocular chemical burns (Thanikachalam et al., 2011). Ocular surface lesions secondary to paraquat and treated with AM showed decreased complications; the symblepharon appearance rate with AM was 0–34% vs. 87.5% with conventional drug therapy (DT) and the mean corneal epithelial defect closure time was 9.8 ± 3.6 days in the AM group, and 18.2 ± 5.2 days in the DT group (Wang et al., 2015). AM transplantation following excision of the primary pterygium has been shown to decrease the recurrence rate to 2% in post-operative patients with a 12-month follow-up (Katbaab et al., 2008) and provide a recurrence rate of less than 10% in recurrent pterygium (Solomon et al., 2001a; Ma et al., 2005). Comparing the outcomes of a limbal conjunctival autograft (LCAG) with cryopreserved AM graft to treat recurrent pterygium, no differences were found in terms of the healing time of the epithelial defect, the degree of conjunctival inflammation, or the frequency of complications such as punctate epithelial keratitis, episcleral fusion, corneal pannus, and delayed healing of the corneal epithelium (Chen et al., 2017; Pan et al., 2018) (Figure 4). The use of cryopreserved AM following reconstructive ocular surface surgery after the removal of conjunctival and limbal tumors can achieve complete healing of the lesion, with a smooth and stable surface, free of scarring and symblepharon appearance (Asoklis et al., 2011; Palamar et al., 2014; Goktas et al., 2017). Treating bacterial, herpetic, neurotrophic, post-surgical, rheumatoid arthritis, and persistent epithelial defect corneal ulcers with frozen AM achieves early healing of the corneal epithelial wound in cases refractory to conventional treatment (Lee and Tseng, 1997; Acar, 2020; Schuerch et al., 2020). The use of AM on the descemetocele showed stability of the ocular surface, increased thickness of the thinned surface, and adequate integration of the ocular stroma when evaluated by high-resolution optical coherence tomography (Sultana et al., 2018). Cryopreserved, fresh and freeze-dried AM transplantation in Stevens Johnson syndrome/toxic epidermal necrolysis has been shown to reduce ocular inflammation, promote epithelialization, maintain visual acuity, and prevent the generation of symblepharon and ocular scarring (Muqit et al., 2007; Pruet et al., 2014; Gervasio and Wu, 2015; Chen. Z et al., 2021; Nassim et al., 2021). Ocular lesions secondary to immune diseases (Sjögren’s syndrome, Mooren’s ulcer, peripheral ulcerative keratitis, rheumatoid arthritis, atypical hemolytic uremic syndrome, and others) with or without ulcerations or corneal perforations that have been treated with cryopreserved AM have shown a decrease in recurrence, a increase in re-epithelialization, a decrease in the inflammatory process, and maintained visual acuity (Ngan and Chau, 2011; Jia et al., 2014; Cheng et al., 2018; Shafer et al., 2019; Acar, 2020; Mishra et al., 2020; Chen. Z et al., 2021). Surgical techniques for ocular placement of AM for different ocular pathologies have been described; these techniques increase the therapeutic benefits obtained after the use of AM (John, 2003; Malhotra and Jain, 2014).

## Other surgical applications

Other surgical areas have used AM for therapeutic purposes, but on a more limited basis; however, the results of its use have had positive outcomes. Oral cavity surgeries cover a wide range of medical and dental problems. The use of cryopreserved AM for periodontal soft tissue healing after dental implant surgery has been effective in the first 3 weeks after surgery, through decreased pain and epithelial growth, adhesion, and migration (Velez et al., 2010). Vestibuloplasty for preprosthetic treatments and resection of extensive mucosal lesions in the gingival and alveolar area occasionally result in bone exposure. However, the use of hyper dry AM in these procedures resulted in pain relief, good hemostatic status, adequate width of keratinized tissue, stabilization of dentures and a higher survival rate of dental implants (Tsuno et al., 2014). In lyophilized form, it has been used to cover extensive intraoral excisions after resection of precancerous lesions, improving the re-epithelialization of lesions and reducing pain in patients (Hazarika et al., 2022). The dry AM graft in post-tonsillectomy as a biological dressing reduced postoperative pain, facilitated rapid return to normal diet and promoted the wound healing process (Faramarzi et al., 2021). The use of decellularized and lyophilized amnion/chorion membrane in patients with laryngeal squamous cell carcinoma, who develop pharyngocutaneous fistula after preoperative chemotherapy, radiotherapy, and total or extended laryngectomy, demonstrated a median complete wound healing time of 18 days (Kakabadze et al., 2016). Surgical areas such as urology have used AM for therapeutic and regenerative purposes, such as the placement of dehydrated amnion/chorion membrane around the neurovascular bundle (NVB) during robotic-assisted laparoscopic prostatectomy (RARP). AM led to the recovery of urinary continence at 8 weeks in 81% of the AM group and 74.1% of the control group. Sexual potency was restored at 8 weeks in 65.5% of patients treated with AM and 51.7% of the control group (Patel et al., 2015). Moreover, frozen AM has been used for the reconstruction of extensive ureteral wall defects secondary to ureteral strictures, demonstrating by ultrasound the absence of obstruction and normal width of the ureters, low recurrence rate of strictures, residual hydronephrosis, and urinary tract infections (Koziak et al., 2007). These are some examples of how AM has been used in other surgical areas, although the surgical application of placental tissue has not yet been deepened.

## Products of the amniotic membrane on the market

To date, there exists a great variety of amniotic membrane-based products (Table 1). Some of them are enriched with growth factors (AmnioTechnology, 2017; Surgilogix, 2020; Mimedx, 2021; Aedicell, 2022; Integra, 2022), cytokines (Mimedx, 2021; Aedicell, 2022; Integra, 2022), extracellular matrix proteins (AmnioTechnology, 2017; Mimedx, 2021; Biotissue, 2022; Integra, 2022), chorion (INTEGRA, 2022), umbilical cord, or collagen (BIOLOGICS, 2022; BIOTISSUE, 2022). The AM presentations are dehydrated or cryopreserved. Some products have health approval, and each one has its indications for use, but in general, only placed on wounds in sterile form.

**TABLE 1:** Current amniotic membrane products (Features and indications).

Name of the product or the brand	Features	Approval	Indication	References
Amniofix^®^ (MiMedx)	Dehydrated amniotic membrane + EGF, KGF, HA, IL-6	FDA/AATB	-Chronic wounds	Fetterolf and Snyder, (2012); Mimedx, (2021)
			-Debridement	
			-Amputations	
			-Dehiscence	
			-Decubitus ulcers	
			-Trauma	
			-Pilonidal cysts	
			-Burns	
EpiFix^®^ (MiMedx)	Dehydrated amniotic membrane + EGF, KGF, HA, IL-6	FDA/AATB	-Healing defects	Fetterolf and Snyder, (2012); Mimedx, (2021)
			- Diabetic food ulcers	
			-Varicose ulcers	
			-Decubitus ulcers	
			-Wounds	
			-Desbridement	
SXBarrier^®^ (SurgiLogix)	Cryopreserved amniotic membrane + PDFF-AA, PDGF-BB, bFGF, TGF-β1, EGF, FGF, VEGF	FDA	-Wounds	Surgilogix, (2020)
			-Surgical Incision	
			-Tissue regeneration	
Surgraft^®^ (Surgenex)	Dehydrated amniotic membrane	FDA	-Diabetic food ulcers	Surgenex, (2021)
			-Wounds	
			-Food injuries	
			-Orthopedical surgeries	
Biovance^®^ (Alliqua Biomedical/Celularity)	Decellularized Dehydrated amniotic membrane	FDA	- Non-infected partial-thickness wounds	BioMedical, (2020)
			-Chronic wounds	
			-Diabetic food ulcers	
			-Pressure ulcers	
			-Venous ulcers	
			-Surgical wounds	
			-Burns	
			-Trauma wounds	
Clarix^®^1K (Amiox/BioTissue)	Cryopreserved amniotic membrane from umbilical cord	FDA/AATB/ISO	-Complex Bone and Joint Reconstruction ---Soft Tissue Repair and Reconstruction	Morkin and Hamrah, (2018); Biotissue, (2022)
			-Nerve Repair and Decompression	
			-Joint Arthroplasty and Arthrodesis	
			-Cartilage Repair	
			-Fractures and Non-unions	
			-Traumatic Wounds and Reconstruction,	
			-Surgical Wound Healing	
			-Dehiscence	
Clarix^®^100 (Amiox/BioTissue)	Thinner cryopreserved amniotic membrane	FDA/AATB/ISO	-Minimally Invasive Achilles	Morkin and Hamrah, (2018); Biotissue, (2022)
			-Midfoot/Forefoot Fractures	
			-Tendon/Nerve Repair	
			-Ganglion Cyst Excision	
			-Bunionectomy	
			-Cheilectomy	
Neox^®^1K (Amiox/BioTissue)	Cryopreserved ultra-thick amniotic membrane	FDA/AATB/ISO	Diabetic Foot Ulcers	Morkin and Hamrah, (2018); Biotissue, (2022)
			-Chronic Wounds	
			-Venous Leg Ulcers	
			-Arterial Ulcers	
			-Pressure Ulcers	
			-Wound Dehiscence	
			-Burns	
Neox^®^100 (Amiox/BioTissue)	Cryopreserved amniotic membrane	FDA/AATB/ISO	Diabetic Foot Ulcers	Morkin and Hamrah, (2018); Biotissue, (2022)
			-Chronic Wounds	
			-Dehisced Wounds	
			-Granulating/Epithelializing Wounds	
			-Hypertrophic Scars/Keloids	
			-Non/Minimally Exudating	
			-Wounds	
			-Pressure Ulcers	
			-Venous Ulcers	
			-Burns.	
-Neox®RT (Amiox/BioTissue)	Cryopreserved amniotic membrane	FDA/AATB/ISO	Diabetic Foot Ulcers	Morkin and Hamrah, (2018); Biotissue, (2022)
			-Chronic Wounds	
			-Venous Leg Ulcers	
			-Arterial Ulcers	
			-Pressure Ulcers	
			-Wound Dehiscence	
			-Burns	
-Prokera^®^ (Amiox/BioTissue)	Cryopreserved amniotic membrane + cleared	FDA/AATB/ISO	-Keratitis	Morkin and Hamrah, (2018); Biotissue, (2022))
			-Corneal scars	
			-Chemical burns	
			-Corneal defects	
			-Partial limbal stem cell deficiency	
			-Inflammatory ocular surface diseases	
-Plurivest^®^ -Demavest (Aedicell)	Cryopreserved amniotic membrane and γ ray irradiated + TGF 1, HGF, PDFG-BB, PIG-F, SDF-1, VEGF, TIMP 1, 2, IL-4,6,8,10	FDA/AATB	-Partial and Full Thickness	AediCell, (2022)
			-Wounds	
			-Drainage Wounds	
			-Trauma Wounds (abrasions, lacerations, and skin tears)	
			-Second Degree Burns	
			-Diabetic Ulcers	
			-Pressure Ulcers	
			-Venous Ulcers	
			-Chronic Vascular Ulcers	
			-Surgical (donor sites/grafts post-surgery, post-laser surgery, podiatric)	
AmnioClear (LiventaBioscience)	Amniotic membrane	FDA	-Wounds	Liventabioscience, (2014)
			-Diabetic food ulcers	
-AmnioBioGraft^®^ (Alamo Biologics)	Amniotic membrane single	FDA/AATB	-Regenerative medicine	Biologics, (2022)
			-Wound management	
			-Chronic and non-healing dermal wounds	
			-Cutaneous wound care	
			-Reconstructive medicine	
			-Ocular injuries and reparative eye work	
			-Burn Care	
-AmnioBioGraft +^®^ (Alamo Biologics)	Amniotic membrane dual layer	FDA/AATB	-Regenerative medicine	Biologics, (2022)
			-Wound management	
			-Chronic and non-healing dermal wounds	
			-Cutaneous wound care	
			-Reconstructive medicine	
			-Ocular injuries and reparative eye work	
			-Burn Care	
-AmnioBioGraft Cord^®^ (Alamo Biologics)	Amniotic membrane from Umbilical cord	FDA/AATB	-Regenerative medicine	Biologics, (2022)
			-Wound management	
			-Chronic and non-healing dermal wounds	
			-Cutaneous wound care	
			-Reconstructive medicine	
			-Ocular injuries and reparative eye work	
			-Burn Care	
-XWRAP^®^ (Applied Biologics)	Amniotic membrane	FDA	- Minimize scarring at surgical injury sites	Biologics, (2016)
			- Protection of nerves and tendons at injury sites	
-Allowrap^®^ (Allosource)	The amniotic membrane of double layer	FDA	-Reduction of spinal adhesions	Sanders and Annest, (2018)
			-Trauma	
			-Sports medicine	
-Amnioshield^®^ (αtec)	Dehydrated amniotic membrane	FDA	-Reduce scars	αtec, (2022)
			-Chronic wounds	
			-Healing promotion	
-PalinGen^®^ (AmnioTechnolog)	Amniotic membrane + collagen + Growth factors + Extracellular matrix proteins	FDA/AATB	-Wounds	AmnioTechnology, (2017)
			-Reduce scars	
			-Diabetic food ulcers	
-AmnioExcel^®^	Amniotic membrane + Corion + Extracellular matrix proteins + Growth factors + Cytokines	FDA	-Wounds	Integra, (2022)
-AmnioExcel Plus^®^				
-Omnigraft^®^ (Integra Bioscience)				

FDA, food and drug administration; AATB, american association of tissue bank; ISO, international organization for standardization; IL, Interleukin; VEGFA, Vascular endothelial growth factor A; HA, Hyaluronic acid; HGF, Hepatocyte growth factor; EGF, Epidermal growth factor; FGF, Fibroblast growth factors; PIG-1, Serine/threonine-protein phosphatase 1; PDGF, Platelet-derived growth factor; KGF, Keratinocyte growth factor; NGF, Nerve growth factor; TGF, Transforming growth factor; TIMP, T cell immunoglobulin mucin protein; SDF, Stromal cell-derived factor.

## Good practices for the clinical use of AM

To get the benefits of AM, we have to attend some recommendations for its use: a) store as the manufacturer indicates on each product. b) place AM to epithelial side up, unless you want to limit the angiogenic process. c) Maintain sterile conditions. d) AM must be in direct contact with the lesion. e) Do not place any substance on the lesion or AM. Failure to heed these recommendations may affect the effectiveness of its use. All AM products are generated under Food and Drug Administration (FDA) criteria and some also under American Association of Tissue Banks (AATB) and International Organization for Standardization (ISO) criteria; this ensures proper processing of AM free of infections or substances that may endanger the health of patients. Today, no contraindications or side effects have been reported for the use of AM.

In addition, the histocompatibility of AM permits the desired functions without generating any local or systemic adverse response in the tissue or tissue cells. The cellular components (hAECs and hAMCs) of AM are considered immune privileged cells and showed remarkable characteristics of low immunogenicity (Insausti et al., 2010; Miki, 2016). This condition is due to a low expression of major histocompatibility class I antigen (HLA-A/B/C) and no expression of major histocompatibility class II antigen (HLA-DR) and β2 microglobulin (Chiavegato et al., 2007; Murphy et al., 2010; Srinivasan et al., 2020); likewise, they do not express HLA-A/B/C costimulatory molecules such as CD80, CD86, and CD40 (Wolbank et al., 2007; Pratama et al., 2011) As such, when hAECs and hAMSCs have been transplanted intravenously into humans, they did not result in hemolysis, allergic reactions, toxicity, or tumor formation (Yang et al., 2018). Also, when AM was transplanted under the skin, it did not elicit a host immune response (Miki and Strom, 2006). All this suggests that the risk of rejection of AM is minimal and makes it a good candidate for therapeutic use.

## Concluding remarks

The therapeutic use of AM has diversified over the years, since the beginning of the 20th century. However, the therapeutic potential of placental tissue has not been fully exploited. Among the advantages of AM is that it does not generate a rejection response and that it degrades days after placement in the tissue. Therefore, once the biosafety of the placental tissue is guaranteed by ruling out the presence of transmissible infections and its sterilization, AM can be used for any desired purpose with complete safety. The information available on AM favors the generation of new clinical trials assessing the therapeutic or regenerative applications of this tissue, with the aim of reducing the presence of sequalae and at the same time trying to improve the clinical, histological, functional, or aesthetic aspects of lesions after the use of AM.

The aforementioned molecular mechanisms and correlating the different therapeutic properties (immunomodulation, anti-scarring, pro-angiogenic/anti-angiogenic, antimicrobial and tissue regeneration). We propose immunomodulation, anti-scarring and tissue regeneration as therapeutic axes of this placental tissue as the main therapeutic effects of AM. These functions are achieved by the structural, protein and cellular components that AM possesses, all of which gives it ideal and necessary characteristics to use this tissue therapeutically. These three central functions are the minimum and most important in the resolution of most diseases. In most of the clinical trials in which AM was used, it demonstrated a positive impact after its use, was safe and did not trigger adverse effects. Thus, AM can be part of the necessary supplies to perform various surgical procedures, in order to increase the success rate of the different surgeries and, at the same time, improve the functional and aesthetic aspects of patients. The integration of the therapeutic effects of AM, the molecular mechanisms and the clinical benefits offered by the amnion will favor more proposals for its use in other surgical techniques, pathologies or surgical areas (Figure 3).

The surgical areas described previously (gynecology, plastic surgery, gastroenterology, traumatology, neurosurgery, ophthalmology, otorhinolaryngology, urology, and dentistry) which have used AM pre-operatively, trans-operatively, or post-operatively, demonstrated shortening in the post-operative recovery time, a reduction in the scarring process, analgesia, a reduction in the inflammatory process, reduced injury recurrence, fewer wound infections, improvements to the tissue microenvironment at the injury site, and the functional and structural recovery of tissues. The clinical improvements that are obtained after the surgical use of AM in different lesions are mediated by the modification of different biological processes that together impact the functionality and status of different tissues (Figure 4). AM may be a good therapeutic tool to be used in many surgical procedures; however, new clinical trials to demonstrate its use and functionality are still to be proposed and conducted.
